# Integrating Bulk and Single‐Cell RNA‐Seq Reveals Glycolysis‐Associated Macrophages and Its Related Tumor Subgroup Signatures to Predict Prognosis and Therapy in Clear Cell Renal Cell Carcinoma

**DOI:** 10.1155/humu/3125551

**Published:** 2026-05-13

**Authors:** Yang Li, Yuan Chen, Ling Wang, Shuai Yuan, Ling Wu, Hu Sun, Danqiong Wang, Hongkai Lv

**Affiliations:** ^1^ Department of Geriatric Medicine, Shanxi Bethune Hospital, Shanxi Academy of Medical Sciences, Third Hospital of Shanxi Medical University, Tongji Shanxi Hospital, Taiyuan, China; ^2^ Department of Geriatrics, Tongji Hospital, Tongji Medical College, Huazhong University of Science and Technology, Wuhan, China, hust.edu.cn; ^3^ Pathology Department, Taiyuan Central Hospital, The Ninth Clinical Medical College of Shanxi Medical University, Taiyuan, China; ^4^ Department of Urology, Shanxi Bethune Hospital, Shanxi Academy of Medical Sciences, Third Hospital of Shanxi Medical University, Tongji Shanxi Hospital, Taiyuan, China; ^5^ Tumor Center, Shanxi Bethune Hospital, Shanxi Academy of Medical Sciences, Third Hospital of Shanxi Medical University, Tongji Shanxi Hospital, Taiyuan, China; ^6^ Department of Urology, Shanxi Medical College Seventh Affiliated Hospital: Linfen People′s Hospital, Linfen, China

**Keywords:** clear cell risk model, renal cell carcinoma, machine learning, macrophage, renal cell carcinoma, single-cell genomics

## Abstract

**Background:**

Clear cell renal cell carcinoma (ccRCC) is the most common histological type of RCC and is marked by aggressive nature and poor survival. However, therapeutic options remain limited and yield suboptimal outcomes. Macrophages exhibit marked heterogeneity within ccRCC, exerting a substantial impact on the malignant progression of tumors and resistance to therapeutics.

**Methods:**

This study utilized single‐cell sequencing and transcriptomics to identify a subset of macrophages associated with glycolysis and the most interactive tumor subpopulation, in order to explore the link between macrophages and ccRCC risk. Moreover, employing machine learning techniques, we crafted a precise gene signature to predict patient prognosis, with clinical implications.

**Results:**

We identified a macrophage subpopulation primarily characterized by glycolytic metabolism and a closely associated tumor cell subpopulation, both significantly correlated with poor prognoses in ccRCC patients. Then, we use hdWGCNA to identify key genes and functional modules of cell subgroups, and use 101 types of machine learning methods to establish a prognosis model of six genes: CENPA, ITM2B, TUBA1B, TNFSF13B, SNX3, and TNNT1 in the RNAseq cohorts of ccRCC patients. Patients in the high‐risk group exhibited poorer prognoses, with functional enrichment indicating the presence of modules associated with malignant progression. Additionally, immune infiltration analysis revealed higher levels of immune cell infiltration in this group, suggesting their potential responsiveness to immunotherapeutic interventions.

**Conclusion:**

Our study introduces a novel, robust ccRCC prognostic model, providing new insights and potential therapeutic strategies for precision treatment of ccRCC patients.

## 1. Introduction

Renal cell carcinoma (RCC) affects over 400,000 people worldwide annually [1]. The incidence of RCC is higher in men, with an average age of diagnosis around 60 years. Clear cell renal cell carcinoma (ccRCC) accounts for approximately 70% of all RCC cases and is characterized by distinct pathological features that pose significant risks to patients′ well‐being [2]. Other types of kidney cancer include chromophobe cell carcinoma, sarcoma, and adenocarcinoma. ccRCC is considered a moderately malignant form of cancer, exhibiting a light yellow appearance with transparent, jelly‐like substances in the pathological section [2]. Early detection is crucial for effective treatment of ccRCC [3]. Surgical intervention remains the primary approach, but patient prognosis is still suboptimal [4]. Therefore, there is an urgent need to explore the molecular mechanisms underlying ccRCC pathogenesis to identify more effective treatment strategies.

The tumor immune microenvironment (TIME) comprises various components, including tumor cells, immune cells, and cytokines [5]. The interactions between these components can either promote or inhibit antitumor immunity [6]. Although the immune system can initially eliminate tumors through the cancer–immune cycle, tumors eventually develop immunosuppressive microenvironments, allowing them to evade immune surveillance [6, 7]. Immunotherapy is aimed at reshaping the TIME and restoring the tumor‐killing ability of antitumor immune cells [8]. Various approaches to tumor immunotherapy exist, including monoclonal antibody therapy, immune checkpoint blockade therapy, adoptive cell therapy, oncolytic virus therapy, and tumor vaccines [9].

Within the tumor microenvironment, some immune cells undergo a “switch” by tumor cells, transforming from tumor‐killing to tumor‐promoting behavior, and tumor‐associated macrophages (TAMs) are one such example [10]. Although macrophages have the potential to kill tumor cells, mediate cytotoxicity and phagocytosis, and activate innate or adaptive lymphocyte‐mediated tumor resistance mechanisms, in the tumor microenvironment, they often contribute to cancer progression and metastasis through various mechanisms [11]. These mechanisms encompass the promotion of cancer cell survival and proliferation, the induction of angiogenesis, and the suppression of both innate and adaptive immune responses. Furthermore, researchers have highlighted the inherent plasticity of macrophages, underscoring their potential to be reprogrammed toward tumor‐suppressive functions [12].

In this study, we integrated single‐cell sequencing data from the GSE178481 dataset to construct a comprehensive cell atlas comprising macrophages, T cells, and B cells. Using pseudotime trajectory analysis, we described the developmental trajectory of metastatic cancer cells at different stages, leading to the identification of a cell subcluster common among patients and closely associated with glycolysis. Further cell–cell communication analysis revealed that the macrophage subpopulation associated with glycolytic metabolism serves as the starting point of tumor development. Building upon this finding, we identified a tumor cell subpopulation exhibiting a high‐intensity interaction with glycolytic‐associated macrophages.

Subsequently, we established a high‐low‐risk group based on hub genes and evaluated its clinical applicability. Overall, this study provides valuable insights into the diagnosis and treatment of ccRCC, presenting a promising clinical protocol with potential application value.

## 2. Materials

### 2.1. Public Data Sources

The public scRNA‐seq dataset was downloaded from the Gene Expression Omnibus database [13], with Accession Number GSE178481. Moreover, the bulk RNA‐seq expression dataset and corresponding clinical information of TCGA‐KIRC ccRCC were downloaded from the Genomic Data Commons (GDC) Data Portal.

### 2.2. Single‐Cell RNA‐Seq Data Preprocessing

Cells were subjected to additional quality control measures to ensure high data quality. Specifically, cells were filtered based on the following criteria: (1) a minimum of 300 detected genes, (2) mitochondrial gene percent within the range of 0%–15%, (3) hemoglobin gene percentage within the range of 0–0.1, and (4) a minimum of 3 ribosomal gene expressions. Subsequently, a process of data integration was performed using the CCA (canonical correlation analysis) method.

### 2.3. Spatial Transcriptomics (ST) Data Processing

For the ST (ST‐seq) data analysis, we employed the Seurat R package (Version 4.1.1). The data underwent log‐normalization for standardization, as described by Yaugel‐Novoa et al. [14]. Principal component analysis (PCA) was performed using the RunPCA function. Subsequently, we applied the FindNeighbors and FindClusters functions to identify clusters of similar ST spots. Preliminary annotations of distinct clusters were conducted using SPOTlight (Version 1.7.2), a nonnegative matrix factorization (NMF)–based spatial deconvolution framework. To visualize the expression patterns of identified genes, we utilized the SpatialFeaturePlot function in Seurat.

### 2.4. Functional and Pathway Enrichment Analysis

Gene set functional analyses were conducted using the “clusterProfiler” and “GSVA” R packages [15]. The GSVA analyses incorporated gene sets from Gene Ontology (GO), Kyoto Encyclopedia of Genes and Genomes (KEGG), and Reactome pathway databases. Additionally, Hallmark gene sets and Reactome gene sets were sourced from the “msigdbr” R package [16].

### 2.5. Pseudotime Analysis

A single‐cell pseudotime trajectory was constructed using the R package “monocle3” (Version 1.0.1) [17]. UMAP (Uniform Manifold Approximation and Projection) method was employed to reduce dimensions, and the “plot_cells” function was used for visualization. To screen for differentially expressed genes (DEGs), the “graph_test” function was utilized, with the threshold for the Morans index set at > 0.3 and the *q*‐value (corrected *p* value) threshold set at < 0.001.

### 2.6. Cell–Cell Communication Analysis

In this study, we conducted cell–cell communication analysis using the “CellChat” R package Version 1.1.3 [18]. From each cell subcluster, we randomly selected 500 cells using the “subset” function. The analysis was performed using the CellChat database, which includes information on “secreted signaling,” “ECM‐receptor,” and “cell–cell contact” interactions. To ensure the reliability of the results, we filtered out any interactions involving cell types with a count of fewer than 10 cells.

### 2.7. Survival Analysis

We identified the Top 10 marker genes for each cell subcluster and calculated subcluster feature scores for 537 ccRCC patients from TCGA using GSVA. By combining this data with overall survival (OS) time, we performed a Kaplan–Meier survival analysis using the “survival” R package.

### 2.8. HdWGCNA Analysis

A high‐dimensional weighted gene coexpression network analysis (hdWGCNA) was constructed at the single‐cell level using the R package “hdWGCNA.” The threshold for achieving a scale‐free topology model fit greater than 0.85 was set, and a soft threshold of 8 was chosen to optimize connectivity. The TCGA cohort was then scored with modules using GSVA. To assess the correlations between modules and phenotype, the Spearman test was employed. Furthermore, a protein–protein interaction (PPI) network was generated using the “HubGeneNetworkPlot” function.

### 2.9. Differential Expression Analysis

Differential expression analysis was performed using the “DESeq2” R package. Genes were identified as differentially expressed if they exhibited a fold change greater than 2‐fold (log2FoldChange > 1 or log2FoldChange < −1) and a statistical significance with an adjusted *p* value less than 0.05.

### 2.10. Signature Generated From Machine Learning–Based Integrative Approaches

To achieve a robust and accurate consensus model, we combined 10 different machine learning algorithms and 101 algorithm combinations. The integrated algorithms encompassed various techniques such as random survival forest (RSF), elastic network (Enet), Lasso, Ridge, stepwise Cox, CoxBoost, partial least squares regression for Cox (plsRcox), supervised principal components (SuperPC), generalized boosted regression modeling (GBM), and survival support vector machine (survival‐SVM). The integrated algorithms encompassed 10 base algorithms: RSF, Enet, Lasso, ridge, stepwise Cox, CoxBoost, plsRcox, SuperPC, GBM, and survival‐SVM. These were systematically paired (e.g., RSF + Lasso and RSF + StepCox) to generate 101 unique integrative prediction models. The final risk score was computed as a linear combination of the six hub genes weighted by their Cox regression coefficients, and patients were divided into high‐ and low‐risk groups according to the median risk score. All analyses were performed in R Version 4.2.2 unless otherwise specified.

### 2.11. Drug Sensitivity Prediction

We utilized the R package “pRRophetic” to predict drug sensitivity for commonly used or potential chemotherapy drugs in ccRCC. The TCGA cohort, comprising 537 patients, was stratified into two groups using the median of gene module GSVA scores.

### 2.12. Cell Lines and Cell Culture

The human normal renal proximal tubular epithelial cell line HK‐2 (RRID: CVCL_0302) and human clear cell renal carcinoma cell lines 786‐O (RRID: CVCL_1051) and 769‐P (RRID: CVCL_1050) were used in this study. HK‐2 cells were obtained from the American Type Culture Collection (ATCC), and 786‐O and 769‐P cells were purchased from the Cell Bank of the Chinese Academy of Sciences (Shanghai, China). HK‐2 cells were cultured in DMEM/F12 medium (Gibco, Cat#11320033) supplemented with 10% fetal bovine serum (FBS; Gibco, Cat#10099141C) and 1% penicillin–streptomycin (Gibco, Cat#15140122). 786‐O and 769‐P cells were maintained in RPMI‐1640 medium (Gibco, Cat#11875093) supplemented with 10% FBS and 1% penicillin–streptomycin. All cells were cultured at 37°C in a humidified incubator with 5% CO_2_ and routinely tested for mycoplasma contamination.

### 2.13. Quantitative Real‐Time PCR

Total RNA was extracted from cells using TRIzol reagent (Invitrogen, Cat#15596026) according to the manufacturer′s instructions. RNA concentration and purity were measured using a NanoDrop 2000 spectrophotometer (Thermo Scientific). A total of 1‐*μ*g RNA was reverse‐transcribed into cDNA using a reverse transcription kit (Takara, Cat#RR047A). qPCR was performed using SYBR Green Master Mix (Applied Biosystems, Cat#A25742) on a QuantStudio 5 Real‐Time PCR System (Applied Biosystems). The reaction mixture (20 *μ*L) consisted of 10‐*μ*L SYBR Green Master Mix, 0.4‐*μ*L forward primer (10 *μ*M), 0.4‐*μ*L reverse primer (10 *μ*M), 2‐*μ*L cDNA template, and 7.2‐*μ*L RNase‐free water. The amplification conditions were as follows: initial denaturation at 95°C for 10 min, followed by 40 cycles of 95°C for 15 s and 60°C for 60 s. GAPDH was used as an internal control, and relative gene expression levels were calculated using the 2^−*ΔΔ*Ct^ method with HK‐2 cells as the reference group. All experiments were performed in triplicate. The primer sequences used in this study were as follows:

GAPDH‐F: GACAGTCAGCCGCATCTTCT

GAPDH‐R: GCGCCCAATACGACCAAATC

CENPA‐F: GGCCCTATTGGCCCTACAAG

CENPA‐R: AAAGTCCAGACAGCATCGCA

ITM2B‐F: CCTCAGTCCCATTCCCCAAC

ITM2B‐R: GAAGGCGTTTGCATCTGCTT

TUBA1B‐F: TATGGCTGCCCTTGAGAAGGA

TUBA1B‐R: GCAGGGCCAAAAGGAATGGAT

TNFSF13B‐F: TTTGAACCACCAGCTCCAGG

TNFSF13B‐R: TGCAATCAGTTGCAAGCAGTC

SNX3‐F: AAGCCGCAGAACCTGAATGA

SNX3‐R: GTTTGCGGGTTGCTCACATC

TNNT1‐F: CTCCATCACAGCCCTCCTG

TNNT1‐R: TTTCAGCTTCGCCATCAGGT

### 2.14. AI Use Statement

We confirm that no artificial intelligence–generated content (AIGC) tools, such as ChatGPT and other large language models (LLM) tools, were used in the writing or preparation of this manuscript. All content and text in this paper were created solely by us.

## 3. Results

### 3.1. Single‐Cell RNA Sequence Data Integration and Clustering

**Figure 1:**
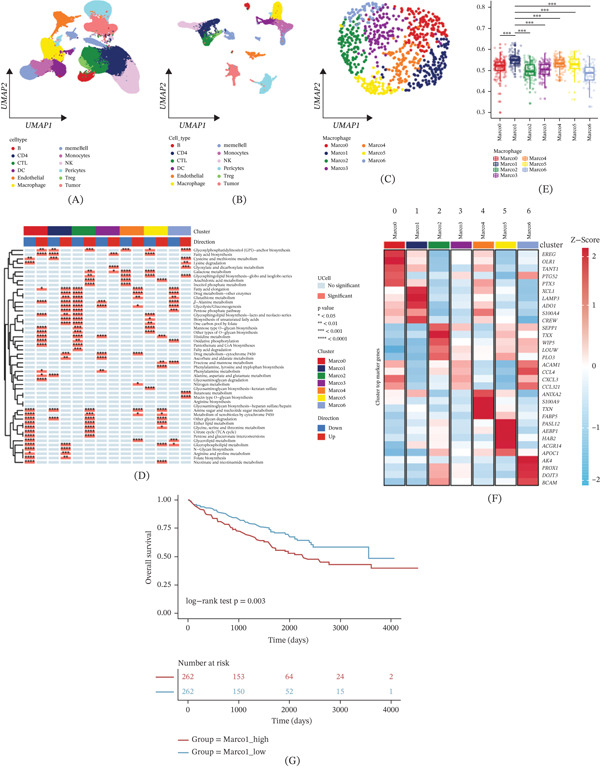
scRNA‐seq profiling of the ccRCC. (A, B) UMAP plot of all the single cells from 25 ccRCC samples, 152,218 cells total. (C) Macrophages from single cells were isolated for clustering. (D, E) Ucell algorithm assesses KEGG metabolism‐related pathways. (F) Heatmap of Top 5 marker gene expression in macrophage subpopulation. (G) The K‐M curve.

Initially, we employed CCA to cluster renal clear cell carcinoma within the GSE178481 dataset. Subsequently, we translated the single‐cell data into metacells, identifying 12 distinct cell types, including B cells, CD4 cells, macrophages, DC cells, among others (Figure 1A,B). The macrophages were further subdivided into seven unique clusters (Figure 1C). To gain insights into the metabolic pathways, we utilized the Ucell algorithm to assess KEGG metabolization‐related pathways. Remarkably, the Marco1 subgroup exhibited the highest glycolysis score (Figure 1D,E), with comparatively lower activity in oxidative phosphorylation and other metabolic pathways across macrophage subclusters. Examining the expression of Top 5 marker genes, we found strong correlations with specific cell types (Figure 1F). Moreover, we applied the ssGSEA method to evaluate the scores of 18 marker genes associated with Marco1 in the TCGA‐ccRCC dataset. Based on the median Marco1 score, patients were grouped and subjected to survival analysis. The results demonstrated a significant correlation between Marco1 and patient prognosis, with higher Marco1 scores indicating poorer patient outcomes (Figure 1G).

### 3.2. hdWGCNA Identifies the Hub Genes of Marco1 Associated With Immunity

**Figure 2:**
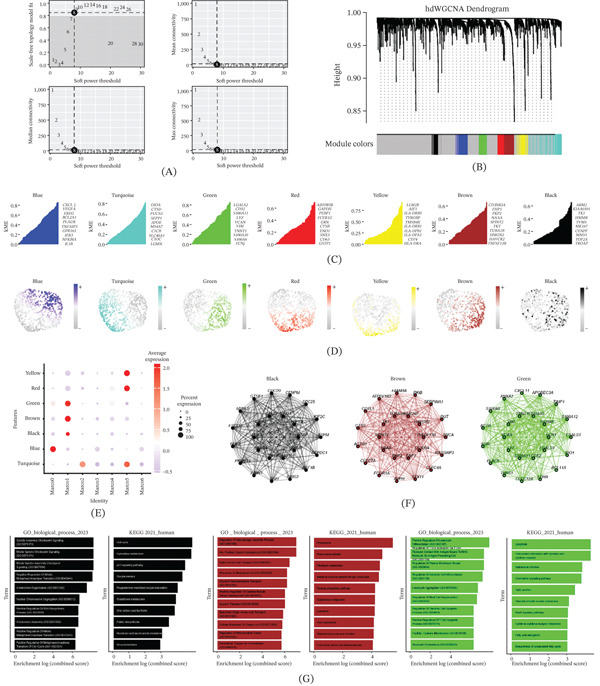
hdWGCNA identifies the hub genes. (A) hdWGCNA soft threshold (Select 8). (B) Seven gene modules were identified. (C) Top 10 genes for each module. (D) Ucell score for each module. (E) Scores of individual modules in macrophage subsets. (F) Protein–protein interaction. (G) GO‐BP and KEGG enrichment of each module.

Subsequently, we conducted hdWGCNA to uncover the predominant molecular characteristics of macrophages. A soft threshold of 8 was applied to construct a scale‐free network, ensuring optimal connectivity and resulting in the identification of 7 distinct gene modules (Figure 2A–C). To focus on the specific molecular signatures associated with Marco1, Ucell scores were calculated for these modules in macrophage subsets. Interestingly, three modules—green, brown, and black—exhibited the highest scores in Marco1 and were chosen for further investigation (Figure 2D,E). To understand the biological functions of each module, we conducted PPI analysis of the Top 25 genes within these three modules. Notably, CDC20, CTSS, and RRM2 emerged as central hub genes (Figure 2F). To gain insights into the functional implications, we performed GO and KEGG enrichment analyses for these three modules. The results revealed strong associations with immunosuppression and other pertinent functions, including viral protein interaction with cytokine and cytokine receptor, intestinal immune network for IgA production, and *Staphylococcus aureus* infection (Figure 2G).

In conclusion, our study delved into the gene expression modules specific to Marco1, successfully identifying key hub genes contributing to immunosuppression and shedding light on the underlying biological mechanisms.

### 3.3. Pseudotime Trajectory Reconstruction and Cell–Cell Communication Analysis With Macrophage Subpopulation

**Figure 3:**
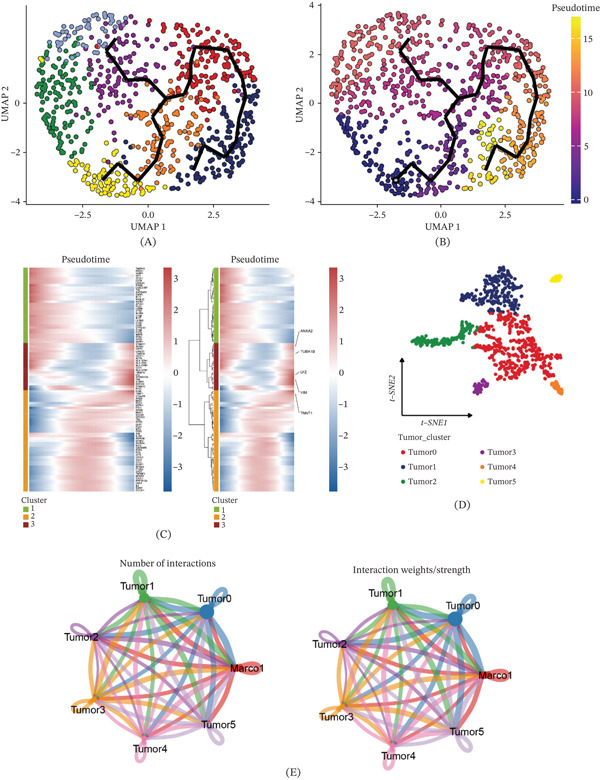
Pseudotime analysis and cell–cell communication analysis. (A, B) Pseudotime analysis. (C) Gene heatmaps of pseudotime changes. (D) Clusters of individual tumor cells. (E) Phase relationship analysis.

Although cell clustering is effective for recognizing subtypes, capturing the progression of cell states in continuous biological processes presents challenges. To address this, we employed pseudotime trajectory analysis to infer the lineage relationships among the macrophage subsets. This method allows us to estimate the developmental progression of cells and infer their potential transitional paths along the trajectory. Pseudotime analysis revealed that the macrophage subsets associated with glycolytic metabolism reached a developmental endpoint (Figure 3A). Furthermore, considering the developmental time perspective, these subsets also exhibited a developmental endpoint (Figure 3B). This endpoint likely represents a terminally polarized state promoting tumor progression. We then visualized gene heatmaps depicting time‐dependent changes (Figure 3C), which highlighted the significance of ANXA2, TUBA1B, LYZ, VIM, and TNNT1 genes in the occurrence and progression of ccRCC. Next, we performed individual grouping of tumor cells (Figure 3D) and observed that Tumor0 exhibited the most pronounced intercellular interaction with glycolytic macrophage subsets (Figures 3E and S1A). Additionally, we showed each tumor subcluster′s OS analyses (Figure S1B). This finding indicates a potential functional relationship between Tumor0 cells and the glycolytic macrophage subsets in ccRCC development.

**Figure 4:**
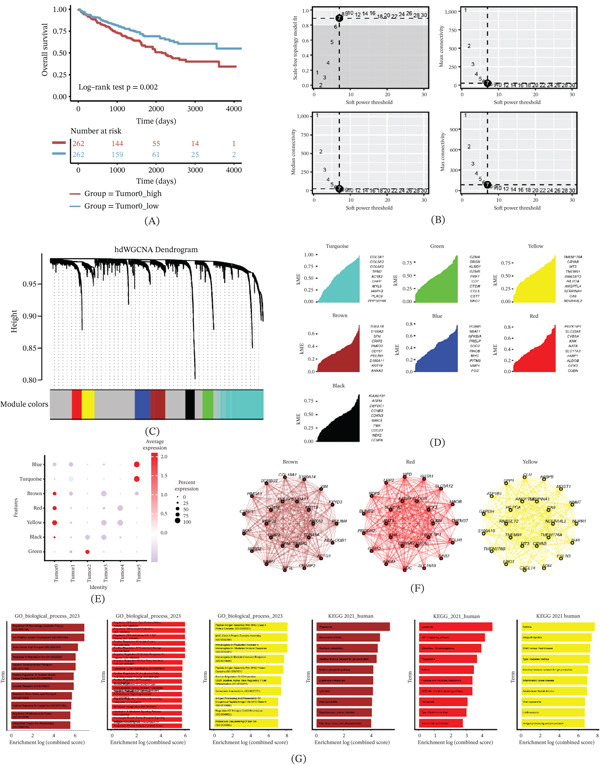
Identification of gene coexpression modules among tumor cells. (A) Tumor0 was negatively associated with OS. (B) hdWGCNA soft threshold (Select 7). (C) Seven gene modules were identified. (D) Top 10 genes for each module. (E) Scores of individual modules in tumor subsets. (F) Protein–protein interaction. (G) GO‐BP and KEGG enrichment of each module.

Afterward, we proceeded to assess the prognostic significance of tumor cell subpopulations. Out of the six subclusters identified from the patient samples, only one subcluster exhibited a significant association with patient prognosis. Notably, Tumor0 showed a negative correlation with OS time, as determined by the log‐rank test (*p* = 0.002) (Figure 4A). No significant prognostic associations were observed for other tumor subclusters in the overall cohort. A soft threshold of 7 was applied to construct a scale‐free network, ensuring optimal connectivity and resulting in the identification of 7 distinct gene modules (Figure 4B–D). To focus on the specific molecular signatures associated with tumor, Ucell scores were calculated for these modules in tumor subsets. Interestingly, three modules—red, brown, and yellow—exhibited the highest scores in Tumor0 and were chosen for further investigation (Figure 4E). To understand the biological functions of each module, we conducted PPI analysis of the Top 25 genes within these three modules. Notably, VIM, GPX3, and SPP1 emerged as central hub genes (Figure 4F). To gain insights into the functional implications, we performed GO and KEGG enrichment analyses for these three modules. The results revealed strong associations with macrophage apoptosis and other pertinent functions, including neutrophil activation, phagosome, HIF‐1 signaling pathway, and glycolysis/gluconeogenesis (Figure 4G).

In summary, our analysis revealed that among the identified subclusters, only Tumor0 displayed a significant impact on OS, suggesting its potential as a prognostic marker in ccRCC patients.

### 3.4. Integrative Construction of a Consensus Signature

**Figure 5:**
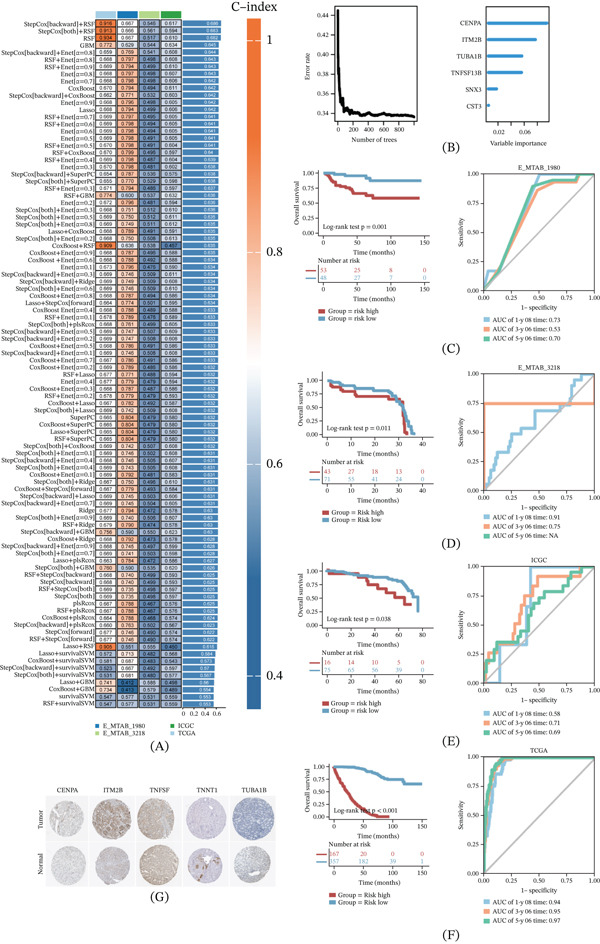
Machine learning–based construction of a prognostic model using genes from glycolytic macrophages and subpopulations of relevant tumor cells. (A) A total of 101 kinds of prediction models. (B) Random forest. (C‐F) KM curve and 1‐, 3‐, and 5‐year ROC curve. (G) IHC images from the Human Protein Atlas (HPA) database.

In the TCGA dataset, we employed 101 prediction models using 10‐fold cross‐validation to calculate the C‐index for each model across all validation datasets (Figure 5A). Interestingly, the optimal model was a combination of StepCox (backward) and RSF with the highest average C‐index (0.686), outperforming all other models in all validation datasets. The hub genes, determined by their coefficients in the random forest algorithm, underwent stepwise Cox proportional hazards regression, resulting in the identification of a final set of six hub genes (Figure 5B). Although hub genes such as CDC20, CTSS, and RRM2 were identified in macrophage modules, the Final 6‐gene signature (CENPA, ITM2B, TUBA1B, TNFSF13B, SNX3, and TNNT1) emerged from the highest performing integrative models, reflecting optimal prognostic robustness in bulk RNA‐seq cohorts. These genes are associated with metabolism and are implicated in the occurrence and development of ccRCC. A risk score was calculated for each patient using a Cox model, considering the expression of the six hub genes weighted by their regression coefficients. The survminer package helped determine the optimal cutoff value for stratifying patients into high‐ and low‐risk groups. Furthermore, we validated the risk score′s prognostic value in three independent external cohorts (ICGC and MTAB‐3218), which are completely independent of the TCGA training set, to assess OS time in ccRCC patients belonging to the high‐risk and low‐risk groups. The results consistently demonstrated a significantly lower OS rate in the high‐risk group compared with the low‐risk group (Figure 5C–F). Furthermore, we obtained IHC images from the Human Protein Atlas (HPA) database (Figure 5G).

In conclusion, our study leveraged advanced modeling techniques and hub gene analysis to establish a robust prognostic risk score for ccRCC patients, showcasing its potential as a valuable tool for predicting patient outcomes.

### 3.5. Expression Pattern of Identified Genes in ST‐Seq Dataset

**Figure 6:**
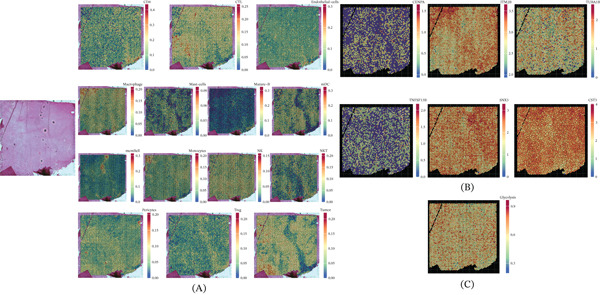
Expression profiling of six genes in ST‐seq datasets. (A) Deconvolution of stRNA‐seq data. (B) Offering a visual representation of the expression profiles of the six identified genes within the stRNA‐seq dataset. (C) GSVA analysis.

To gain a more comprehensive understanding of the expression patterns of the six identified prognostic genes, we meticulously examined their expression profiles in the ST RNA sequencing data. Initially, we performed cell type annotation on the ST‐seq data using a NMF‐based spatial deconvolution framework to infer the cell‐type composition of each spot (Figure 6A). The spatial expression patterns of the six hub genes are presented in Figure 6B, demonstrating their heterogeneous but tumor‐enriched distribution across the tissue section. Furthermore, GSVA analysis revealed enhanced glycolysis activity in regions with high gene expression (Figure 6C), suggesting a close association between these prognostic genes and metabolic reprogramming within the tumor microenvironment. The expression patterns of these six genes were consistently observed across both datasets. Importantly, the spatial colocalization of these gene signatures with glycolysis‐high regions suggests a potential physical interaction between macrophages and tumor cells within the tumor microenvironment, with notably higher expression in tumor spots versus normal‐adjacent regions.

### 3.6. Enrichment Analysis of DEGs in High‐ and Low‐Risk Groups

**Figure 7:**
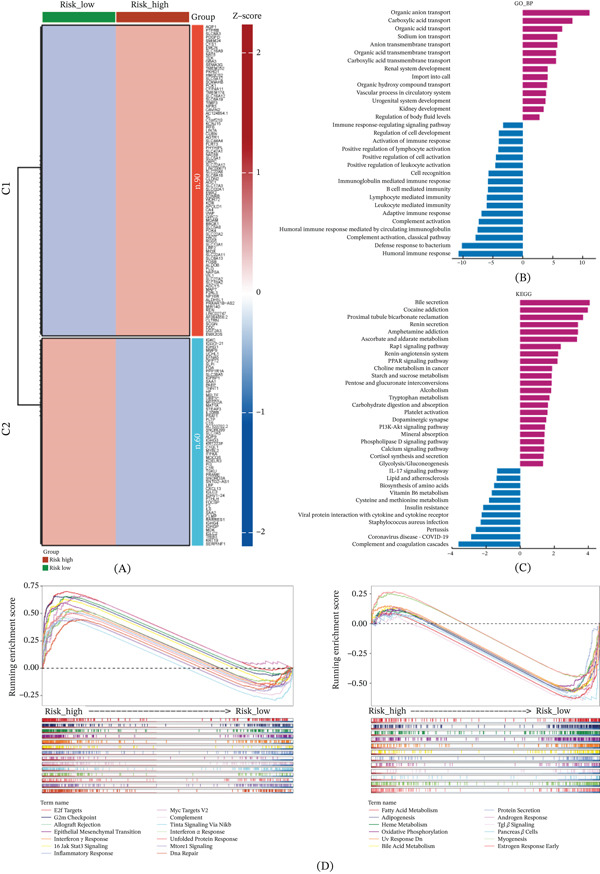
Enrichment analysis of differentially expressed genes in high‐ and low‐risk groups. (A) Differential gene expression heatmap. (B) GO‐BP enrichment analysis of differential genes, purple: upregulated; blue: downregulated. (C). Enrichment analysis of KEGG. (D) GSEA enrichment analysis of Hallmark gene set.

Following the stratification of patients into high‐risk and low‐risk groups based on the optimal cutoff value of the risk score, we conducted differential gene expression analysis, resulting in 90 highly expressed genes and 60 low‐expressed genes (Figure 7A).

Subsequently, we performed GO enrichment analysis on these DEGs. The results revealed upregulated gene enrichment in processes related to organic anion transport, carboxylic acid transport, renal system development, and sodium ion transport. Conversely, downregulated gene enrichment was observed in processes associated with humoral immune response, defense response to bacterium, and humoral immune response mediated by circulating immunoglobulin (Figure 7B). Moreover, KEGG enrichment analysis demonstrated that upregulated genes were significantly enriched in proliferative and metabolic signaling pathways such as the PI3K − Akt signaling pathway, PPAR signaling pathway, and Rap1 signaling pathway. On the other hand, downregulated gene enrichment was observed in immune‐related signaling pathways, including the IL‐17 signaling pathway and coronavirus disease—COVID‐19 pathway (Figure 7C). Additionally, GSEA enrichment analysis on Hallmark gene sets showed significant enrichment of inflammatory response, allograft rejection, and interferon gamma response signaling pathways in the upregulated genes. In contrast, pathways related to adipogenesis, fatty acid metabolism, and heme metabolism were significantly enriched in the downregulated genes (Figure 7D). In summary, our findings suggest that the DEGs in the high‐risk and low‐risk groups are closely associated with the malignant phenotype of tumors, proliferative metabolic pathways, and the immune microenvironment.

### 3.7. Immune Microenvironment Associations With High‐ and Low‐Risk Groups

**Figure 8:**
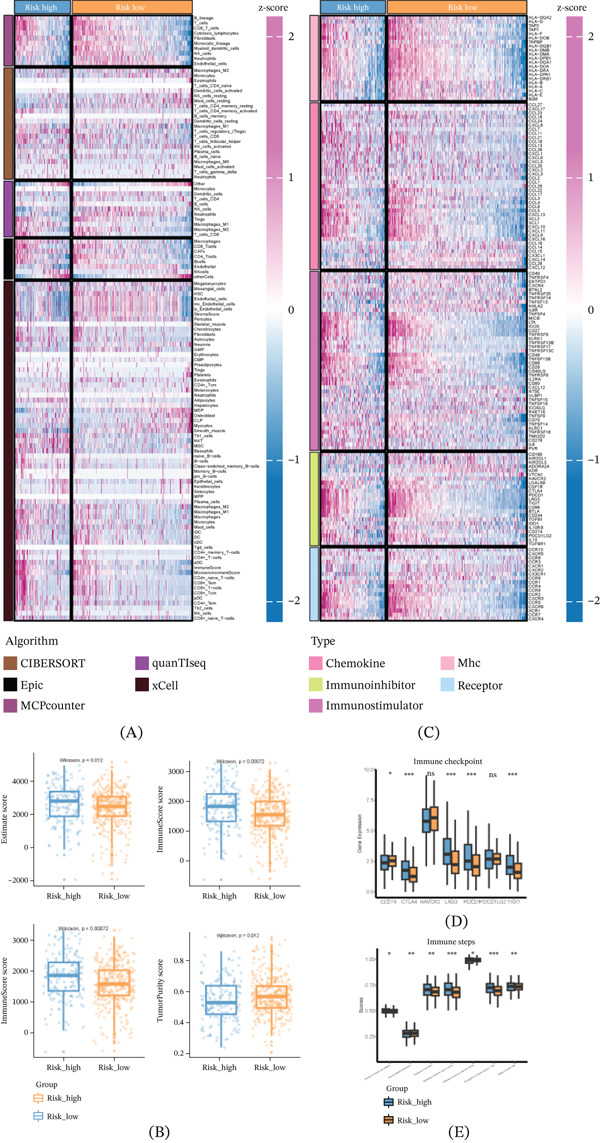
Associations between immune microenvironments and high‐ and low‐risk groups. (A) CIBERSORT, Epic, MCPcounter, quanTIseq, and xCell. (B) Stroma score, tumor purity, and estimate score. (C) Immunomodulators. (D) Immune checkpoints. (E) Immune steps.

Our results revealed consistent infiltration patterns across algorithms, with particularly strong concordance for macrophages and T cell subsets, supporting the reliability of the observed differences between high‐ and low‐risk groups (Figure 8A). Subsequently, the Estimate algorithm was employed to evaluate group immune scores, including stroma score, tumor purity, and estimate score (Figure 8B).

Our results revealed that the high‐risk group exhibited significantly higher estimate score, immuneScore score, and stromalScore, whereas the tumorpurity score was significantly lower compared with the low‐risk group. Furthermore, we analyzed the expression of immunomodulators in the high‐risk and low‐risk groups (Figure 8C), finding a strong association between immunomodulators and tumor risk. Additionally, the high‐risk group showed a significant presence of immune checkpoints (Figure 8D).

Moreover, the immunization procedure score indicated that the high‐risk group had significantly higher scores for release of cancer cell antigens, priming and activation, trafficking of immune cells to tumors, and recognition of cancer cells by T cells compared with the low‐risk group (Figure 8E). In summary, our findings suggest that the high‐risk group of patients may exhibit features indicative of immune rejection.

**Figure 9:**
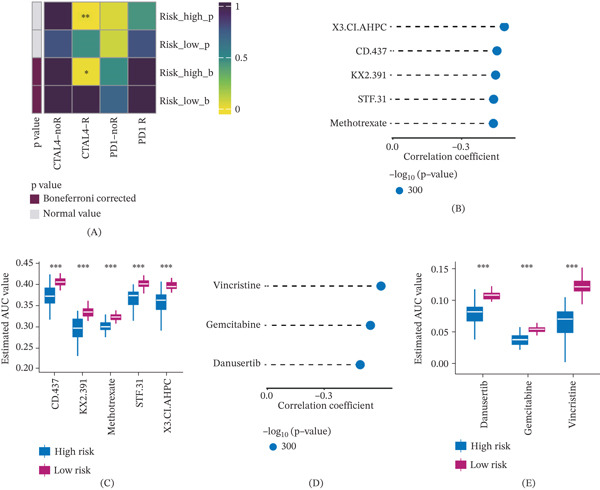
Drug sensitivity prediction. (A) Immunotherapy response. (B, C) Drug sensitivity analysis in the CTRPV2 database. (D, E) Drug sensitivity analysis in the PRISM database.

We employed Submap to predict the immunotherapy response in the high‐risk and low‐risk groups, revealing that CTLA4 immunotherapy had a significant effect on the high‐risk group (Figure 9A). Additionally, we conducted drug sensitivity analysis for the high‐risk group using the CTRPV1 and PRISM databases through the oncopredict package to identify potential therapeutic agents. Compared with the low‐risk group, the high‐risk group was resistant to drugs such as X3.Cl. AHPC, CD.437, KX2.391, STF.31, and methotrexate (Figure 9B,C). In addition, our results demonstrated that chemotherapy agents, including danusertib and vincristine, exhibited higher sensitivity in the low‐risk group compared with the high‐risk group (Figure 9D,E). Both agents have been investigated in preclinical or early clinical studies for RCC, supporting the potential clinical relevance of these predictions. This risk model holds the potential to guide clinical medication and immunotherapy decisions for ccRCC patients and provides valuable insights for personalized treatment approaches. It provides valuable insights for personalized treatment approaches, aiming to improve therapeutic outcomes.

### 3.8. Experimental Validation Results

**Figure 10:**
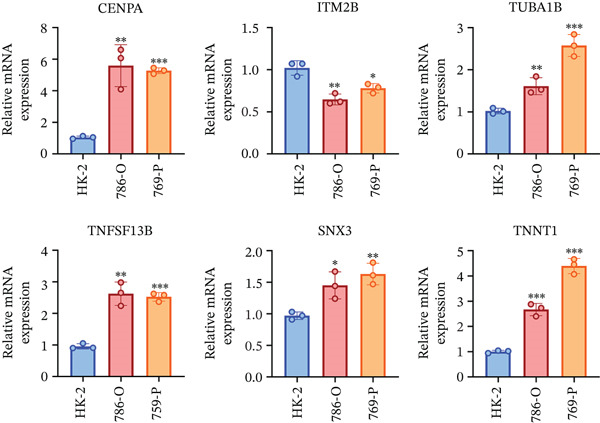
qPCR validation of candidate gene expression in renal cell lines. The mRNA expression levels of CENPA, ITM2B, TUBA1B, TNFSF13B, SNX3, and TNNT1 were examined by quantitative real‐time PCR (qPCR) in normal renal epithelial cells (HK‐2) and renal cell carcinoma cell lines (786‐O and 769‐P). Gene expression levels were normalized to internal control and presented as relative mRNA expression. Data are shown as mean ± SD (*n* = 3). Statistical significance was determined compared with HK‐2 cells.  ^∗^ *p* < 0.05,  ^∗∗^ *p* < 0.01,  ^∗∗∗^ *p* < 0.001.

To experimentally validate the differential expression of the six hub genes (CENPA, ITM2B, TUBA1B, TNFSF13B, SNX3, and TNNT1) identified from the integrated single‐cell and bulk RNA‐seq analyses, we performed quantitative real‐time PCR (RT‐qPCR) in the normal renal tubular epithelial cell line HK‐2 and the ccRCC cell lines 769‐P and 786‐O. As shown in Figure 10, all six genes were significantly upregulated in both ccRCC cell lines relative to HK‐2 cells, although the magnitude of increase varied among them. CENPA exhibited the most pronounced elevation (fold change approximately 5.0–5.5,  ^∗∗∗^ *p* < 0.001), followed closely by TNNT1 (fold change 2.8–4.5,  ^∗∗∗^ *p* < 0.001). The remaining four genes—TNFSF13B, TUBA1B, ITM2B, and SNX3—showed moderate yet statistically significant upregulation, with fold changes ranging from 1.4 to 2.8 (*p* values from  ^∗^ *p* < 0.05 to  ^∗∗∗^ *p* < 0.001). These experimental findings are highly consistent with the prognostic risk model derived from TCGA and other cohorts, in which higher expression of these genes was associated with poorer patient outcomes. The upregulation observed in 786‐O and 769‐P cells further supports the biological relevance of the glycolysis‐associated macrophage‐derived signature in ccRCC progression and provides direct wet‐lab validation beyond in silico analyses.

## 4. Discussion

In the initial phase of this study, we employed clustering techniques to categorize ccRCC samples based on specific immune cell types. Considering the significant role of macrophages in tumor immunity, they became the primary focus of our research [19]. Subsequently, we conducted a more refined subgroup analysis exclusively targeting macrophage subtypes. This led us to perform KEGG enrichment analysis to explore the functional aspects associated with these distinct macrophage subsets, revealing a strong association with glycolytic pathways, known to be pivotal in tumor development [20]. Further analysis highlighted the macrophage subgroup Marco1 as closely linked to glycolysis. Importantly, patients with higher Marco1 scores within the Marco1 subgroup experienced poorer outcomes, suggesting its potential as a prognostic indicator for ccRCC in the future. The primary aim of this study was to identify significant gene modules in glycolytic‐associated macrophages using hdWGCNA. We initially identified seven modules and subsequently selected the three most relevant modules for functional enrichment analysis based on Ucell scores. Numerous studies have emphasized the impact of inhibiting tumor immunity on the occurrence and development of ccRCC [21, 22]. Therefore, we further investigated the subsets of ccRCC tumor cells with the strongest interaction with glycolytic‐associated macrophage subsets using quasitime series analysis and intercellular communication analysis. Interestingly, we found that one of the Top 10 genes in this tumor subgroup exhibited a strong association with the prognosis of ccRCC. Lastly, we established a prognostic model using machine learning methods to provide a valuable reference model for the prognosis diagnosis of ccRCC.

Next, we identified five hub genes (ANXA2, TUBA1B, LYZ, VIM, and TNNT1) known to be involved in critical metabolic responses in tumors [23–27]. For instance, TNFSF13B is critical for B cell activation and linked to antitumor immunity, whereas CENPA overexpression is often associated with cell cycle progression. The inclusion of these genes reflects a balance between metabolic activity and immune regulation. Subsequently, we fitted 101 prediction models using the LOOCV framework, distinguishing between high‐risk and low‐risk groups. Interestingly, the DEGs in the high‐low risk group were enriched in essential tumor immune and metabolism signaling pathways, such as humoral immune response, defense response to bacterium, humoral immune response mediated by circulating immunoglobulin, PI3K − Akt signaling pathway, PPAR signaling pathway, and Rap1 signaling pathway [14, 28]. These findings suggest that the activation or inactivation of these pathways could play a pivotal role in ccRCC development, offering potential therapeutic targets for ccRCC treatment [29, 30].

The immune microenvironment significantly impacts tumor immune escape. To explore differences in the immune microenvironment between high‐ and low‐risk groups, we employed five algorithms (CIBERSORT, Epic, MCPcounter, quanTIseq, and xCell) to evaluate infiltrating cells in the tumor microenvironment. Our results indicated an immune‐inflamed but immunosuppressive microenvironment in the high‐risk group, which may contribute to tumor immune escape in ccRCC.

Finally, we investigated the sensitivity of the high‐low risk group to immunotherapy drugs. Notably, alisertib, gemcitabine, and vincristine demonstrated better therapeutic effects on the high‐risk group, and CTLA4 emerged as a potential target for ccRCC immunotherapy [31].

To further strengthen the clinical relevance of our six‐gene prognostic signature, we performed experimental validation using RT‐qPCR in the normal renal tubular epithelial cell line HK‐2 and two ccRCC cell lines (769‐P and 786‐O). Consistent with the bioinformatics findings, all six hub genes (CENPA, ITM2B, TUBA1B, TNFSF13B, SNX3, and TNNT1) were significantly upregulated in both ccRCC cell lines compared with HK‐2 cells (Figure 10). These results provide direct wet‐lab evidence supporting the upregulation of the signature genes in malignant renal cancer cells and offer valuable insights for future clinical translation in ccRCC. Nevertheless, as this study is based on retrospective public datasets, potential cohort bias and batch effects cannot be completely excluded, which may affect the generalizability of the findings.

In summary, this study integrated single‐cell sequencing data and transcriptomics to develop a macrophage‐associated tumor risk model and analyze its clinical relevance. The established ccRCC risk model provides potential immunotherapeutic targets and drug options for ccRCC, offering valuable insights for clinical treatment strategies. Given the association of the high‐risk group with enhanced immune infiltration and checkpoint expression, targeting glycolytic reprogramming in TAMs may represent a promising synergistic strategy with immune checkpoint inhibitors in ccRCC. However, a limitation of this study is that the drug sensitivity results are based on computational predictions from public databases. Future experimental validation is necessary to confirm the therapeutic efficacy of these identified agents.

## 5. Conclusions

In this study, our primary objective was to identify a distinct subset of macrophages with a specific emphasis on glycolytic metabolism and their most relevant tumor subpopulation for intercellular interactions. Subsequently, we are aimed at developing gene feature signatures closely associated with both entities, enabling accurate prognostic predictions for patients. Leveraging 101 diverse machine learning algorithms, we generated the most performant gene signature and pinpointed six core genes (CENPA, ITM2B, TUBA1B, TNFSF13B, SNX3, and TNNT1). These genes demonstrated correlations with patient prognosis and immune infiltration. We firmly believe that further exploration of these genes, implicated in the interplay between macrophages and tumors, will yield a more profound understanding of the malignant progression in ccRCC. The multiple combinations of machine learning algorithms offer a promising avenue for uncovering novel biological markers in ccRCC, thus contributing to enhanced strategies for diagnosis and treatment.

## Author Contributions

Yang Li: conceptualization, methodology, and writing—original draft preparation. Yuan Chen and Ling Wang: data curation and formal analysis. Shuai Yuan and Ling Wu: investigation and validation. Hu Sun and Danqiong Wang: software and visualization. Hongkai Lv: conceptualization, supervision, funding acquisition, writing—review and editing, and project administration.

## Funding

This study was supported by the Central Guidance Fund for Local Science and Technology Development (YDZJSX2025D073) and General Programs of Shanxi Provincial Health Commission (2024024).

## Disclosure

All authors have read and agreed to the published version of the manuscript.

## Conflicts of Interest

The authors declare no conflicts of interest.

## Supporting information


**Supporting Information** Additional supporting information can be found online in the Supporting Information section. Figure S1: The Tumor0 exhibited the most pronounced intercellular interaction with glycolytic macrophage subsets. (A) Analysis of the relationship between tumor cell subsets and glycolytic macrophage subsets. (B) Kaplan–Meier curve.

## Data Availability

The single‐cell RNA sequencing data analyzed in this study were derived from the publicly available dataset GSE178481 in the Gene Expression Omnibus (GEO) database. The bulk RNA sequencing data and corresponding clinical information were obtained from The Cancer Genome Atlas Kidney Renal Clear Cell Carcinoma (TCGA‐KIRC) cohort, available through the GDC Data Portal. All datasets used are openly accessible in the public domain.
