# Supplementary figures and images for: Integrating Bulk and Single‐Cell RNA‐Seq Reveals Glycolysis‐Associated Macrophages and Its Related Tumor Subgroup Signatures to Predict Prognosis and Therapy in Clear Cell Renal Cell Carcinoma
Source: Hum Mutat. 2026 May 13;2026:3125551. doi: 10.1155/humu/3125551 (PMC13171712; doi:10.1155/humu/3125551)

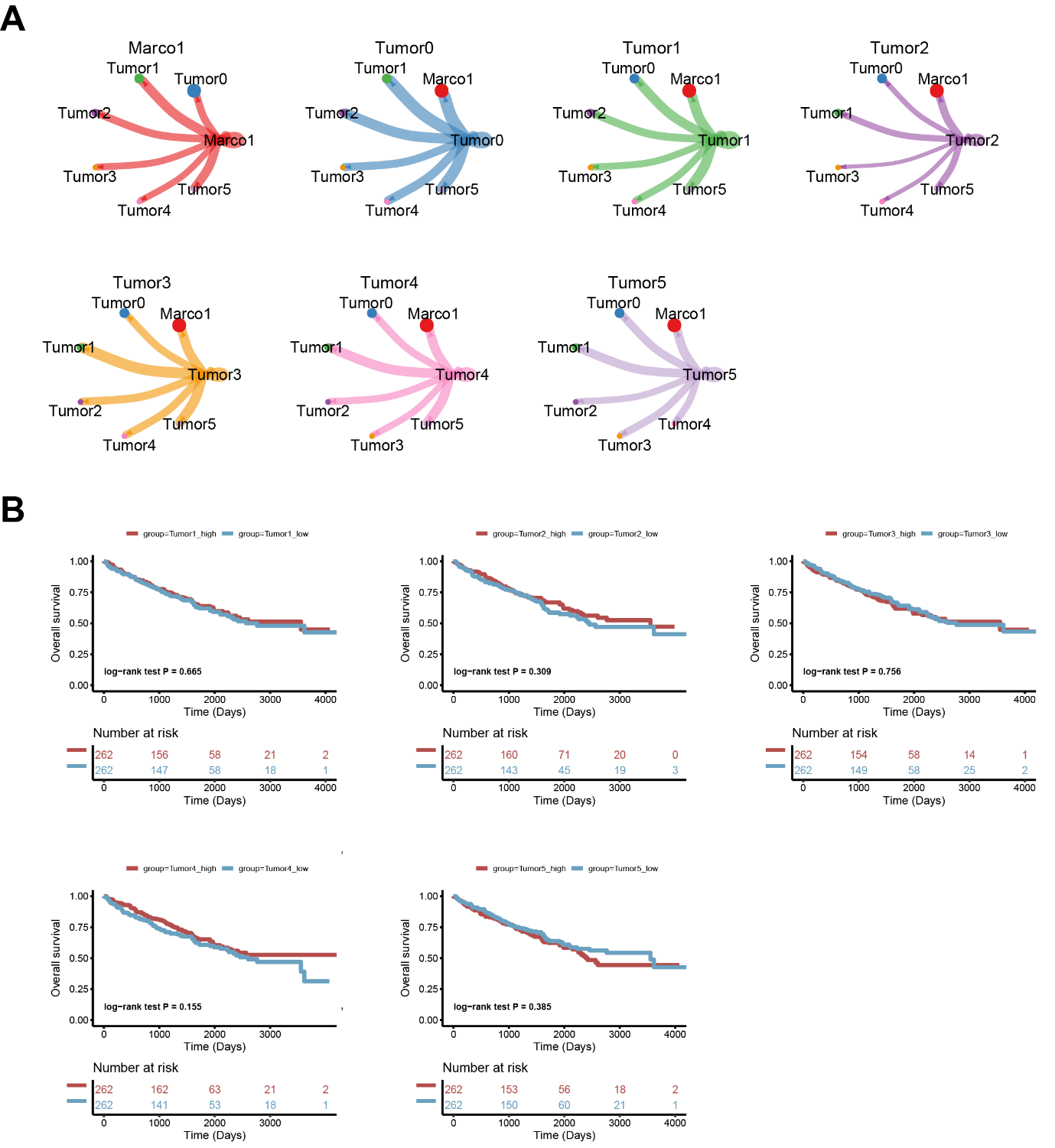

Supplement: Supplementary file 1 — Supporting Information Additional supporting information can be found online in the Supporting Information section. Figure S1: The Tumor0 exhibited the most pronounced intercellular interaction with glycolytic macrophage subsets. (A) Analysis of the relationship between tumor cell subsets and glycolytic macrophage subsets. (B) Kaplan–Meier curve. [file HUMU-2026-3125551-s001.tiff]
